# A Novel Myosin Essential Light Chain Mutation Causes Hypertrophic Cardiomyopathy with Late Onset and Low Expressivity

**DOI:** 10.1155/2012/685108

**Published:** 2012-04-11

**Authors:** Paal Skytt Andersen, Paula Louise Hedley, Stephen P. Page, Petros Syrris, Johanna Catharina Moolman-Smook, William John McKenna, Perry Mark Elliott, Michael Christiansen

**Affiliations:** ^1^Department of Clinical Biochemistry and Immunology, Statens Serum Institut, 2300S Copenhagen, Denmark; ^2^Department of Biomedical Sciences, University of Stellenbosch, Cape Town, South Africa; ^3^The Heart Hospital, University College London Hospital, London, UK

## Abstract

Hypertrophic cardiomyopathy (HCM) is caused by mutations in genes encoding sarcomere proteins. Mutations in *MYL3*, encoding the essential light chain of myosin, are rare and have been associated with sudden death. Both recessive and dominant patterns of inheritance have been suggested. We studied a large family with a 38-year-old asymptomatic HCM-affected male referred because of a murmur. The patient had HCM with left ventricular hypertrophy (max WT 21 mm), a resting left ventricular outflow gradient of 36 mm Hg, and left atrial dilation (54 mm). Genotyping revealed heterozygosity for a novel missense mutation, p.V79I, in *MYL3*. The mutation was not found in 300 controls, and the patient had no mutations in 10 sarcomere genes. Cascade screening revealed a further nine heterozygote mutation carriers, three of whom had ECG and/or echocardiographic abnormalities but did not fulfil diagnostic criteria for HCM. The penetrance, if we consider this borderline HCM the phenotype of the p.V79I mutation, was 40%, but the mean age of the nonpenetrant mutation carriers is 15, while the mean age of the penetrant mutation carriers is 47. The mutation affects a conserved valine replacing it with a larger isoleucine residue in the region of contact between the light chain and the myosin lever arm. In conclusion, *MYL3* mutations can present with low expressivity and late onset.

## 1. Introduction

**Figure 1:**
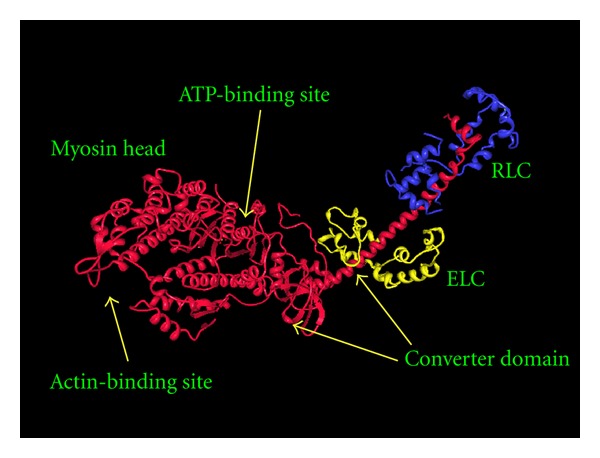
The structure of the myosin S1 fragment (red) with the essential (ELC) (yellow) and regulatory (RLC) (blue) light chains marked. The structure is based on the X-ray crystallographic data available in the form of the 2MYS pdb file [43].

Hypertrophic cardiomyopathy (HCM) is an autosomal dominant genetic disease caused by mutations in genes which encode sarcomeric proteins [1–4]. The most frequently affected genes are MYH7 [5], MYBPC3 [6], and TNNT2 [7], coding for the heavy chain of myosin, the myosin-binding protein-C, and troponin T, respectively. More than 200 mutations have been described in these genes. Furthermore, mutations in a number of other genes, for example, mitochondrial genes [8] have been associated with HCM, albeit at a much lower frequency. Among the rare causes of HCM [9] are mutations in *MYL3* which encodes the myosin essential light chain (ELC) of the sarcomere [4, 10–19]. The ELC is located at the lever arm of the myosin head and stabilises this region (Figure 1) through interaction with the IQ1 motif [20, 21] at aminoacid residues 781–810 [22] in beta myosin. The N-terminus of ELC interacts with actin [23]. Although the precise functional role of ELC has not been defined [24], the protein belongs to the EF-hand family of Ca^2+^-binding proteins [25] and appears to be involved in force development and fine tuning of muscle contraction [26, 27]. The phosphorylation of a C-terminal serine residue has recently been shown to be of major significance for cardiac contraction [28] in zebra fish.

**Figure 2:**
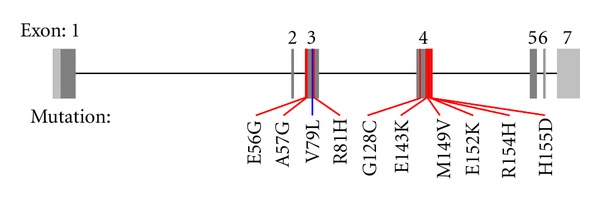
The genomic structure of *MYL3* with the localization of known disease causing mutations indicated.

To date, nine HCM-causing mutations have been described in *MYL3.* Three missense mutations, p.E56G, p.A57G, and p.R81H, have been found in exon 3 and six, p.G128C p.E143K, p.M149V, p.E152K, p.R154H, and p.H155D in exon 4 [4, 10–19] (Figure 2).

The described phenotypes of ELC mutations vary considerably [24], from HCM with an autosomal dominant inheritance and sudden death events, as in p.A57G [13, 17] and p.M149V [10], to a recessive phenotype where only homozygosity was associated with sudden cardiac death [14]. The penetrance is high in middle-aged mutation carriers [24].

Here, we describe a family with a novel *MYL3* mutation characterised by low expressivity and late onset of disease.

## 2. Materials and Methods

The proband and family members were subjected to a full clinical evaluation including family history, physical examination, echocardiography, stress test, and 12-lead electrocardiograph (ECG). Disease status for the proband and family members was determined using conventional diagnostic criteria [29, 30]. All family members gave informed consent for genetic testing, while for testing of children, consent was obtained from the parents.

Genomic DNA was extracted from blood samples using a QIAamp DNA purification kit (Qiagen, Germany). DNA from the proband was screened for mutations in the coding regions of *MYH7, MYBPC3, TNNT2, TPM1, TNNI3, MYL3, MYL2, ACTC, TCAP,* and *CSRP3,* and exons 3, 7, 14, 18, and 49 of *TTN*, as detailed in a previous study [5]. The primers and conditions used for screening *MYL3* have previously been described [9]. Three-hundred ethnically matched Caucasian controls were used to establish frequencies of genetic variants.

Polyphen2 (http://genetics.bwh.harvard.edu/pph2/) was used to predict the effect of the identified ELC variant (p.V79I) [31].

The modelling of ELC and regulatory domain of MYH7 was performed using PDB Protein Workshop (http://www.rcsb.org/) and the PdB file 1WDC [32]. Homology studies and gene-structure studies were performed using BioEdit vers 7.0.9 [33] and Ensembl (http://www.ensembl.org/).

## 3. Results

### 3.1. Family History

**Figure 3:**
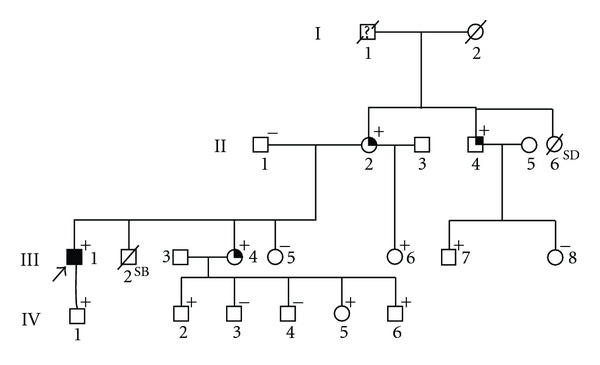
The pedigree of the examined family, the proband is marked by arrow. He is the only clinically affected (black symbol), + indicates family members who are heterozygous for the p.V79I mutation, − indicates family members who were genetically assessed and found to be nonmutation carriers. The three family members with borderline HCM are marked by a black quadrant. SB: still birth. SD marks a 19-year-old girl who suddenly died. No further information was available as to the cause of death.

The proband, a 38-year-old obese (BMI = 44) man, was referred for clinical assessment following the identification of a cardiac murmur during routine health check. He was asymptomatic and had never experienced cardiac symptoms, systemic hypertension, or syncope. Echocardiography demonstrated asymmetrical septal ventricular hypertrophy becoming more concentric towards the apex (max WT 21 mm), a resting left ventricular outflow gradient (36 mm Hg) and left atrial dilation (54 mm) (Table 1). Genotyping of 11 sarcomere genes showed him to be heterozygous carrier of a novel missense mutation (c.235G > A), p.V79I, in *MYL3*, which encodes the ELC of the ventricle. PolyPhen2 prediction of the p.V79I mutation indicated this variant to be “possibly damaging”. The family was offered screening for the mutation and clinical evaluation (see Figure 3 for the pedigree and Table 1 for clinical data on family members).

Examination of the family members resulted in the identification of nine additional heterozygous carriers of the p.V79I mutation, none of whom fulfilled the diagnostic criteria for HCM. Three mutation carriers, see Figure 3 and Table 1, exhibited a borderline phenotype, either based on the presence of an angulated septum in combination with T-wave inversion in AVL on the ECG or diastolic dysfunction in association with left axis deviation (LAD) or LAD and QRS deviation on the ECG. One mutation carrier, a 29-year-old male, exhibited intraventricular conduction defect (IVCD) as an isolated abnormality. The three borderline cases were 58, 55, and 36 years of age, whereas the remaining asymptomatic mutation carriers were 3, 8, 11, 17, 23, and 29 years of age. None of the family members shown in the pedigree had experienced cardiac events or sudden death, but the grandfather of the proband (I-1) was said to have died suddenly and unexpected at the age of 55 years; while a sister (II-6) to the mother had died suddenly and unexpected at the age of 19 years. No information was available as to the cause of these deaths that occurred 4-5 decades ago, and no DNA was available for analysis. As none of the mutation carriers, including the patient with HCM, had any symptoms, severe disease expression, or risk markers, the mutation was considered benign. However, all mutation carriers were offered a follow-up evaluation.

The mutation was not found in 300 controls and had not been registered as a variant in the NCBI SNP databases (dbSNP). Furthermore, there were no clinically affected or borderline cases among the nonmutation carriers.

**Figure 4:**
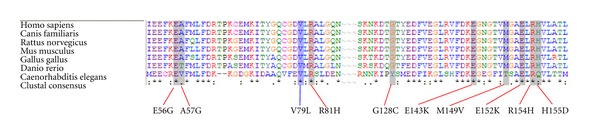
A homology analysis of the known MYL3 mutations, the valine in residue 79 is highly conserved.

**Figure 5:**
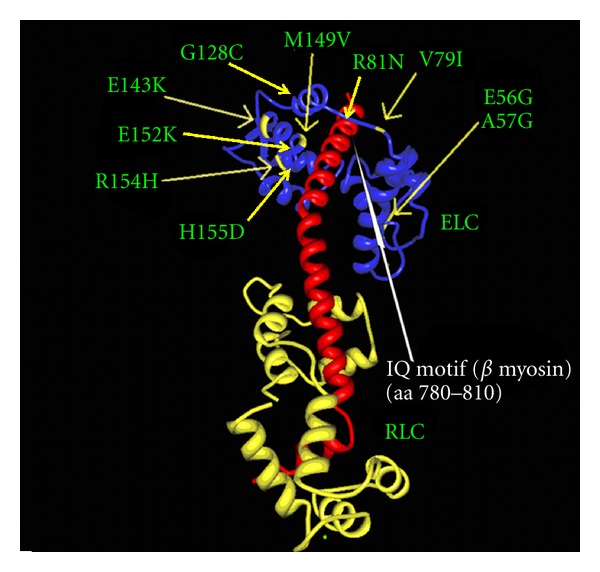
The location of the p.V79I mutation (arrow) in the three-dimensional structure of the regulatory domain of myosin (red) with the essential (ELC) (blue) and regulatory (RLC) (yellow) light chain. The other known mutations in ELC are also marked. It is seen that the p.V79I mutation; as well as the pR81H and p.M149V mutation, is located close to the IQ1 motif of the myosin helix. The IQ1 motif is marked by a white arrow. The figure is based on X-ray crystallographic structure of the myosin myosin regulatory domain of the scallop as given in the 1WDC pdb file [32].

The p.V79I is a novel variant, which is located in exon 3 in the region where two other HCM-associated missense mutations have previously been identified (Figure 2). Multiple species alignment of *MYL3* in seven species indicate that valine is strongly conserved at this position (Figure 4). We examined the location of the p.V79I mutation in the three-dimensional structure of ELC by interpolating the human structure on the structure established for the scallop myosin regulatory domain (Figure 5). The mutation is located on the linker connecting the N- and C-terminal lobes and it is located just two aminoacid residues from the N-C connecting-loop interacting with the first IQ1 motif on myosin (aa 780–810) (Figure 5) [34]. It is possible that the more nonpolar, longer side chain of the isoleucine residue may disrupt interaction of ELC with the positively charged lysine and arginine residues of myosin heavy chain that interface with this ELC region [35].

## 4. Discussion

We have described a patient with mild HCM associated with a novel, dominantly inherited, *MYL3* mutation p.V79I. The mutation segregates with disease and is absent in 600 control alleles in a family in which no other mutation was identified in known HCM-associated genes. The phenotypic presentation of the p.V79I mutation is that of classical HCM with asymmetric septal hypertrophy, just as previously described for the p.A57G mutation in two unrelated Korean families [13, 17] but distinct from the rare midcavitary obstruction previously described in patients with p.E143K [14], p.M149V, and p.R154H mutations [15]. Expression was reduced and/or the disease exhibited late occurrence, as nine mutation carriers were asymptomatic and did not fulfil diagnostic criteria for HCM, and no mutation carriers below 35 years of age were affected or even borderline affected. However, four of the mutation carriers, all >35 years of age, exhibited either a borderline phenotype or ICVD. Strictly defined, the mutation has a penetrance of 10%, but as many of the carriers are below 25 years. If we define the phenotype as borderline HCM with a late onset, we can say that all mutation carriers above the age of 35 exhibit this phenotype. However, a more precise definition of the penetrance of disease will depend on followup studies. Unfortunately, it was not possible to obtain more information about the sudden deaths occurring in the two potential mutation carriers, I-1 and II-6, 4-5 decades ago. However, their existence supports that the mutation carriers are offered clinical follow-up.

The part of beta myosin containing the IQ1 motif that interacts with the N-C-loop of ELC harbours a number of HCM-associated mutations, that are, p.S782D [36], p.S782N [35], p.R787H [4], p.L796F [1], and p.A797T [37], supporting the concept that interference with the interaction between ELC and beta myosin results in HCM. Another part of the beta myosin molecule that comes into close contact with ELC is the arginine residue at position 723 [35], and mutations here also result in HCM, for example, p.R723G [38] and p.R723C [39], which further supports the concept that interference with the interaction between ELC and myosin is a pathogenetic substrate of HCM.

Homology modelling suggests that the p.V79I mutation may interfere with the interaction between ELC and myosin heavy chain, a mechanism which is believed to be the cause of disease for a number of reported mutations. Based on this evidence we find it likely that the p.V79I mutation is disease causing. However, it can not be ruled out that the mutation may only be associated with hypertrophy when other triggering conditions are present. In this case, the proband is very obese; obesity has recently been associated with a cardiac hypertrophic response in mice fed a high-fat diet through inactivation of the Foxo3a transcription factor via the Akt pathway [40]. As the cardiac hypertrophic response in the same mouse model is associated with increased caspase activity [40] and caspase has ELC [41] as its primary substrate in the failing heart, obesity may aggravate the development of a hypertrophic phenotype in conditions with reduced ELC functionality. A less specific aggravation of hypertrophy through the leptin-induced cardiac hypertrophic response seen in neonatal rat cardiomyocytes phenotype could also explain that the proband is the only mutation carrier with clinical HCM [42].

The finding of a clinically silent mutation with low expressivity and late onset raises the question of whether it should entail a detailed followup of mutation carriers. However, as most mutations in *MYL3* have been associated with sudden death [24], it would seem prudent to conduct a clinical followup of mutation carriers. The potential relation between a *MYL3*-based genetic predisposition, the hypertrophic phenotype and obesity in the proband should also strengthen the recommendation to the proband to lose weight.

## Figures and Tables

**Table 1:** 

Pedigree number (Figure 3)	Age/gender	MYL3 genotype	ECG	Echo	Symptoms	Phenotype
III-1 Proband	38/M	p.V79I	LVH and TWI	Asymmetric septal LVH. MWT 21 mm. No RVH. LVOTO (36 mm Hg and LA dilation (54 mm))	None	HCM
II-1	64/M	WT	na	na	None	Unknown
II-2	58/F	V79I	TWI in AVL	Angulated septum	None	Borderline
II-4	55/M	V79I	LAD QRSd	NORMAL	None	Borderline
III-4	36/F	V79I	LAD	Diastolic dysfunction	None	Borderline
III-5	31/F	WT	na	na	None	Unknown
III-6	23/F	V79I	Normal	Normal	None	Normal
III-7	29/M	V79I	IVCD	Normal	None	Normal
III-8	27/F	WT	Normal	Normal	None	Normal
IV-1	11/M	V79I	na	na	None	Unknown
IV-2	17/M	V79I	Normal	Normal	None	Normal
IV-3	15/M	WT	Normal	Normal	None	Normal
IV-4	13/M	WT	na	na	None	Unknown
IV-5	8/F	V79I	Normal	Normal	None	Normal
IV-6	3/M	V79I	Normal	Normal	None	Normal

LVH: left ventricular hypertrophy; MWT: maximum wall thickness; LAD: left axis deviation; IVCD: intraventricular conduction defect; TWI: T-wave inversion; AVL: Augmented unipolar left leadQRSd: QRS deviation; na: not available.
